# Immune checkpoint inhibitor monotherapy is sufficient to promote microenvironmental normalization via the type I interferon pathway in *PD‐L1*‐expressing head and neck cancer

**DOI:** 10.1002/1878-0261.13633

**Published:** 2024-03-21

**Authors:** Jeon Yeob Jang, Bok‐Soon Lee, Mei Huang, Chorong Seo, Ji‐Hye Choi, Yoo Seob Shin, Hyun Goo Woo, Chul‐Ho Kim

**Affiliations:** ^1^ Department of Otolaryngology Ajou University School of Medicine Suwon Korea; ^2^ Department of Biomedical Sciences Ajou University Graduate School of Medicine Suwon Korea; ^3^ Department of Physiology Ajou University School of Medicine Suwon Korea; ^4^ Deparment of Molecular Science and Technology Ajou University Suwon Korea

**Keywords:** head and neck squamous cells carcinoma, immunotherapy, programmed cell death 1 receptor, syngeneic tumor model, tumor microenvironment

## Abstract

Immune checkpoint blockers (ICBs) targeting programmed cell death protein 1 (PD‐1) have been proven to be an effective first‐line therapy against programmed cell death 1 ligand 1 (*PD‐L1*; also known as CD274 molecule)‐expressing head and neck squamous cell carcinoma (HNSCC) in recent KEYNOTE‐048 trial. However, associated changes in the tumor microenvironment (TME) and underlying mechanisms remain elusive. Oral tumors in C57/BL6 mice were induced by administering 7,12‐dimethylbenzanthracene into the buccal mucosa. Single‐cell suspension was isolated from tumor tissue; proliferating cells were injected subcutaneously into the left flank of mice to establish Ajou oral cancer (AOC) cell lines. Subsequently, a syngeneic *PD‐L1*‐expressing HNSCC model was developed by injecting AOC cells into the buccal or tongue area. The model recapitulated human HNSCC molecular features and showed reliable *in vivo* tumorigenicity with significant *PD‐L1* expression. ICB monotherapy induced global changes in the TME, including vascular normalization. Furthermore, the antitumor effect of ICB monotherapy was superior to those of other therapeutic agents, including cisplatin and inhibitors of vascular endothelial growth factor receptor 2 (VEGFR2). The ICB‐induced antitumorigenicity and TME normalization were alleviated by blocking the type I interferon pathway. In summary, ICB monotherapy is sufficient to induce TME normalization in the syngeneic model; the type I interferon pathway is indispensable in realizing the effects of ICBs. Furthermore, these results explain the underlying mechanism of the efficacy of ICB monotherapy against *PD‐L1*‐expressing HNSCC in the KEYNOTE‐048 trial.

## Graphical abstract

The authors show that monotherapy with immune checkpoint blockers (ICBs) targeting PD‐L1 induces global changes in the tumor microenvironment (TME) in head and neck squamous cell carcinoma (HNSCC) in which *PD‐L1* is highly expressed. These results provide mechanistic insights regarding the KEYNOTE‐048 trial that ICB monotherapy can be an effective first‐line therapy in recurrent/metastatic HNSCC.

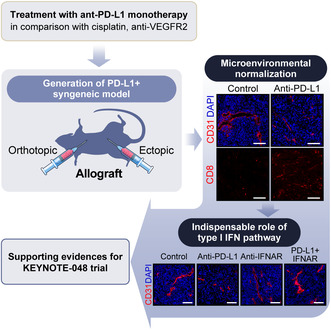

**Abbreviations**
AOC: Ajou Oral Cancer
DEGs: differentially expressed genes
DMBA: 7,12‐dimethylbenzanthracene
EGF: epidermal growth factor
HNSCC: head and neck squamous cell carcinoma
ICB: immune checkpoint blockers
LN: lymph node
PD‐1: programmed death 1
PD‐L1: programmed death‐ligand 1
R/M: recurrent or metastatic
SCC: squamous cell carcinoma
SD: standard deviation
TME: tumor microenvironment

## Introduction

Head and neck cancer is a major cause of morbidity and mortality, with more than 800 000 cases diagnosed annually worldwide [1]. Despite improvements in multimodality treatment protocols, including surgery, radiation, chemotherapy, and targeted therapy, the 5‐year survival rate in advanced head and neck squamous cell carcinoma (HNSCC) remains 40–50% [2]. High mutational burden and tumor‐mediated immune evasion are common features of HNSCC that contribute to conventional treatment failure [3, 4].

In 2016, immunotherapy using anti‐programed cell death protein 1 (PD‐1) inhibitors, such as nivolumab or pembrolizumab, was approved by the US Food and Drug Administration for platinum‐refractory recurrent or metastatic (R/M) HNSCC based on the promising results of large‐scale clinical trials (Checkmate‐041, KEYNOTE‐012) [5, 6, 7]. The degree of PD‐L1 expression is used as a predictive biomarker that has guided different treatment protocols—pembrolizumab in combination with platinum and fluorouracil is used for all patients (biomarker‐unspecified) with R/M HNSCC, and pembrolizumab monotherapy is used for patients with R/M HNSCC, whose tumors express a PD‐L1 combined positive score ≥ 1 [8, 9]. Clinical studies to expand indications and identify biomarkers for immunotherapy have gained increasing interest; however, investigations of the changes in the tumor microenvironment (TME) are insufficient to provide precise mechanistic support for clinical trials.

Given that complex cellular and molecular signals strongly influence TME and the therapeutic responses to immune checkpoint blockers (ICBs) differ across tumor types, an appropriate preclinical model representing specific types of tumors is required to elucidate immunotherapy‐induced TME changes [10]. An appropriate model with a functionally intact immune system is a prerequisite for studying host tumor immune response for immunotherapy research [11]. Syngeneic tumor models of mouse cancer cells implanted into immunocompetent mice have been most commonly utilized in this field; these models, with the advantages of high reproducibility, short latency, and relatively rapid tumor growth, have enabled a higher throughput [11, 12]. In addition, a few studies have reported the use of carcinogen‐induced mouse tumor cell lines to develop a syngeneic HNSCC model [13, 14]. However, the detailed process for establishing a syngeneic HNSCC model has not yet been fully demonstrated. Most researchers lack the use of a syngeneic model system to investigate *in vivo* HNSCC immunotherapy.

In this study, we have refined the previously identified methods of generating murine syngeneic model for HNSCC that recapitulates the tumor immune microenvironment and has reliable tumorigenic potential [13]. Considering these advantages, we further investigated antitumor effects and the changes in TME after ICB monotherapy compared with other chemotherapeutic agents.

## Materials and methods

### Animals

C57BL/6 mice were obtained from Orient Bio Inc. (Seongnam, South Korea). 6–8 weeks‐old male mice were used for all the *in vivo* experiments and provided with high‐pressure steam‐sterilized feed, water, and beddings in the pathogen‐free sterile animal facility. The cages were replaced with clean ones for hygiene purposes twice a week. They were maintained at a temperature 23 ± 1 °C with 50 ± 10% humidity. When handling experimental animals, the experimenter wore dustproof suits, masks, and gloves and used sterilized experimental equipment. All experiments were conducted in accordance with the provisions of the IACUC. This animal study was approved by the Institutional Animal Care and Use Committee of Ajou University (IACUC, 2017‐0062, 2018‐0035).

### Establishment of murine HNSCC cell lines

C57BL/6 mice were administered with 7,12‐dimethylbenzanthracene (DMBA; Sigma‐Aldrich, St. Louis, MO, USA), a carcinogen that induces DNA adduct formation and mutagenic response in mice [15, 16]. DMBA is a well‐known carcinogen showing reliable tumor formation, which exerts similar histopathological and molecular characteristics in mice as in humans [17]. The carcinogen was administered into the buccal mucosa of the oral cavity using either a cotton swab or pipette drop twice a week until overt oral cancer development (27–35 weeks). Tumors were harvested from oral cancer and lymph node (LN) metastasis cases, and a single‐cell suspension was isolated from the tumor tissues. The isolated cells were maintained in DMEM/F12 medium containing 10% fetal bovine serum. Proliferating cells were injected subcutaneously into the left flank of C57BL/6 mice. Three weeks later, single cells were isolated from the tumors of the mice, and the fibroblasts were separated from the cells using a Tumor‐Associated Fibroblast Isolation kit #130‐116‐474 (MACS; Miltenyi Biotec, Bergisch Gladbach, Germany). Finally, we established cancer cell lines called Ajou Oral Cancer (AOC), which were grown in DMEM/F12 media supplemented with 10% fetal bovine serum. Live cells were observed under a light microscope.

### HNSCC syngeneic model and treatment regimen

For the ectopic model, 5 × 10^5^ AOC cells were re‐suspended in 100 μL phosphate‐buffered saline and injected subcutaneously into the flank area. For the orthotopic model, 2 × 10^5^ AOC cells were re‐suspended in 20 μL phosphate‐buffered saline and injected into the buccal or tongue area. After 7 days, the mice were randomly divided into experimental groups, and treatments were initiated. Mice were injected intraperitoneally (i.p.) with anti‐PD‐1 antibody (10 mg·kg^−1^, clone 0F.9G2; BioXCell, Lebanon, NH, USA), anti‐PD‐L1 antibody (10 mg·kg^−1^, clone 10F.9G2; BioXCell), anti‐vascular endothelial growth factor receptor 2 (VEGFR2; 25 mg·kg^−1^, clone DC101; BioXCell), and anti‐interferon‐α/beta receptor (IFNAR; 10 mg·kg^−1^, Clone MAR1‐5A3; BioXCell) twice a week. Cisplatin (5 mg·kg^−1^; Sigma‐Aldrich) was administered i.p. once a week. The optimal dose and schedule of the treatment were determined based on previous studies [18, 19]. Tumor formation was monitored twice a week. Tumor size was measured using a caliper (Mitutoyo Corp., Tokyo, Japan). Volume (mm^3^) was calculated as follows: *V* = *A* × *B* ^2^ × 0.52; *A* and *B* are the longest and shortest superficial diameters, respectively.

### Western blotting

AOC3, AOC3‐LN, AOC11, and AOC11‐LN cell lines were lysed using radioimmunoprecipitation assay (RIPA) buffer (25 mm Tris–HCl pH 7.6, 150 mm NaCl, 1% NP‐40, 1% sodium deoxycholate, and 0.1% SDS; Sigma‐Aldrich). Western blotting was performed using antibodies against p‐EGFR (#2234), EGFR (#2232), p‐ERK (#9101), ERK (#9102), E‐cadherin (#3195), and Zo‐1 (Cell Signaling Technology, Danvers, MA, USA). α‐Tubulin antibody (#CP06; Calbiochem) was used as a loading control.

### Immunocytochemistry

Adherent cells on the coverslips were fixed with 4% paraformaldehyde at room temperature for 15 min. After washing twice with 1X PBS, the cells were incubated with a blocking solution (5% of bovine serum albumin in PBS containing 0.1% Triton X‐100) and then stained with anti‐E‐cadherin or anti‐vimentin (#5741; Cell Signaling Technology) antibodies and incubated at 4 °C overnight. Subsequently, the cells were incubated with secondary antibodies [fluorescein isothiocyanate (FITC) and Cy5]. Nuclei were stained with 4′,6‐diamidino‐2‐phenylindole (DAPI), and images were obtained using a confocal microscope (Zeiss LSM 710; Carl Zeiss, Thornwood, NY, USA).

### Immunohistochemistry

Tumor tissue specimens were fixed with 1% neutral‐buffered formalin (4% paraformaldehyde diluted in 1× PBS), incubated at room temperature for 3 h, dehydrated with 30% sucrose, and stored at room temperature for 3 h. The tissues were embedded in the OCT compound and frozen at −80 °C. Subsequently, the tissue sections were blocked with 5% goat serum in PBST (0.1% Triton X‐100 in PBS) and incubated for 3 h at room temperature with the following primary antibodies: hamster anti‐CD31 (clone 2H8; Millipore, Burlington, MA, USA), rabbit anti‐PD‐L1 (clone 28‐8; BD Biosciences, San Jose, CA, USA), hamster anti‐CD3e (clone 145‐2C11; BD Biosciences), rat anti‐CD8 (clone 53‐6.7; BD Biosciences), rat anti‐CD4 (clone RM4‐5; eBioscience, San Diego, CA, USA), rat anti‐F4/80 (clone BM8; eBioscience), rat anti‐Ly6G (clone 1A8; BD Biosciences), rat anti‐NG2 (clone 546 930; Invitrogen, Carlsbad, CA, USA), rabbit anti‐GLUT1 (clone SA0377; Invitrogen). After several washes with PBS, the tissue sections were incubated for 2 h at room temperature with one or more of the following secondary antibodies: Cy3‐ or FITC‐conjugated anti‐hamster IgG (Jackson ImmunoResearch, West Grove, PA, USA), Cy3‐ or FITC‐conjugated anti‐rabbit IgG (Jackson ImmunoResearch), or Cy3‐conjugated anti‐rat IgG (Jackson ImmunoResearch). Nuclei were stained with DAPI (Invitrogen). The samples were mounted with a fluorescent mounting medium (Dako, Carpinteria, CA, USA). Images were obtained using confocal microscopy (Zeiss LSM 710) and analyzed using Zeiss software.

### Morphometric analysis

Morphometric analyses were performed using imagej software (http://rsb.info.nih.gov/ij) or LSM Image Browser (Carl Zeiss). For vascular and immune cell densities, we measured the CD31^+^ and CD8a^+^ areas, respectively, in 0.1225 mm^2^ fields and presented them as percentages of the total area measured. NG2^+^ pericyte coverage was calculated as the corresponding fluorescent positive length percentage along the CD31^+^ vessels in a random 0.49 mm^2^ field. The hypoxic area was quantified as the percentage of GLUT1^+^ area per random 0.49 mm^2^ field.

### Quantitative measurement for intratumor VEGF‐A protein

Primary tumors were harvested and homogenized in an ice‐cold buffer containing a protease inhibitor cocktail on the indicated days. The VEGF‐A levels were quantitatively measured using the enzyme‐linked immunosorbent assay kit (R&D Systems, Minneapolis, MN, USA) following the manufacturer's instructions.

### RNA‐sequencing (RNA‐Seq) profiling

RNA was isolated from the cells using TRIzol reagent (Invitrogen). For RNA‐seq analysis, mRNA sequencing libraries were prepared using the TruSeq Stranded mRNA Sample Preparation Kit (Illumina, San Diego, CA, USA), according to the manufacturer's protocols. Briefly, mRNA was purified and fragmented from 2 μg total RNA. The libraries were sequenced as paired‐end reads (2 × 100 bp) using NovaSeq 6000 (Illumina). Low‐quality sequence reads with PHRED scores < 30 and adapter sequences were trimmed using the Trim Galore tool and then mapped to the mouse reference genome (mm10). RNA abundance was estimated as the FPKM values using TopHat2/Cufflinks according to the GENCODE database (version M21) with default parameters. Gene expression profiles were normalized using log_2_ transformation and quantile normalization.

### Whole exome sequencing and variant calling

Genomic DNA was fragmented, and exonic regions were captured using the SureSelect XT Mouse All Exon Kit (Agilent, Santa Clara, CA, USA) according to the manufacturer's instructions. Paired‐end sequencing (150 bp) was performed using a NovaSeq 6000 (Illumina). After processing the sequences as described above, they were mapped to mm10 using the Burrows‐Wheeler Alignment tool (BWA‐mem) with default parameters, and the average coverage of the reads was 29.70 × (20.60–48.27). PCR duplicates were identified and removed using Picard (https://broadinstitute.github.io/picard). The base quality scores were normalized using the Genome Analysis Toolkit (GATK). The sequence variations were filtered using the GATK HaplotypeCaller and VariantFiltration under the following conditions: (i) FS > 30, (ii) QD < 2, (iii) QUAL < 50, and (iv) DP < 5, where FS is the Phred‐scaled *P*‐value using Fisher's Exact Test to detect strand bias, QD is variant confidence by depth, QUAL is the base call quality, and DP is the read depth at the variant position. The variants were annotated using the annovar software (https://annovar.openbioinformatics.org), and those in the exome were used in the analysis.

The somatic mutation profile of The Cancer Genome Atlas (TCGA) HNSCC dataset (TCGA‐HNSCC, *n* = 508) was obtained from the NCI's Genomic Data Commons portal (https://portal.gdc.cancer.gov/). For homologous gene mapping, the human gene symbols were converted into the mouse gene symbols using the ‘biomart’ r package (https://bioconductor.org/packages/release/bioc/html/biomaRt.html).

### Gene ontology (GO) and gene set enrichment analyses (GSEA)

GO analysis of the gene sets was performed using david 6.8 software (https://david.ncifcrf.gov). To identify AOC3‐ and AOC11‐specific enriched biological pathways, we obtained 50 HALLMAKR signatures from mSigDB. For each sample, the enrichment score for a gene signature was calculated using the pre‐ranked GSEA method, and the enrichment analysis between sample groups was performed using the GSEA method (https://www.gsea-msigdb.org/gsea).

### Cell viability assay

Cells were seeded at a density of 3 × 10^3^ cells per well in 96‐well plates (Cat#3590, Corning, NY, USA). Cell viability was measured by the CCK‐8 assay (Cat#CK004, Dojindo, Japan) for the indicated periods (24 h, 48 h, 72 h).

### Cell migration assay

8 × 10^4^ cells resuspend in 200 μL serum‐free medium were added in the upper chamber, 500 μL of 10% serum medium was added to the lower chamber. After incubation for 24 h, take out the upper chamber and the cells were fixed with methanol and stained with 0.1% crystal violet (Cat#C0775, Sigma‐Aldrich). Then five random fields were photographed under EVOS M7000 Cell Imaging Systems (×200 magnification, Thermo Fisher Scientific) and the numbers of migrated cells were counted.

### Evaluation of the HPV status

The high‐risk HPV status of each cell was assessed using the PANA RealType™ HPV kit (Panagene Inc., Daejeon, Korea) in accordance with the manufacturer's instructions.

### Statistical analyses

Statistical analyses were performed using the pasw statistics 18 software (SPSS, Chicago, IL, USA). Values are presented as means ± standard deviations. The normal distribution of all datasets was verified using Shapiro–Wilk normality test. Significant differences between groups were determined using Student's *t*‐test or Mann–Whitney *U* test. The significant differences between multiple groups (> 3) were determined using a one‐way analysis of variance or the Kruskal–Wallis test. Results were considered statistically significant at *P* < 0.05.

## Results

### Syngeneic tumor model established using murine HNSCC cells isolated from carcinogen‐induced tumors in the oral cavity and metastatic lymph nodes

**Fig. 1:**
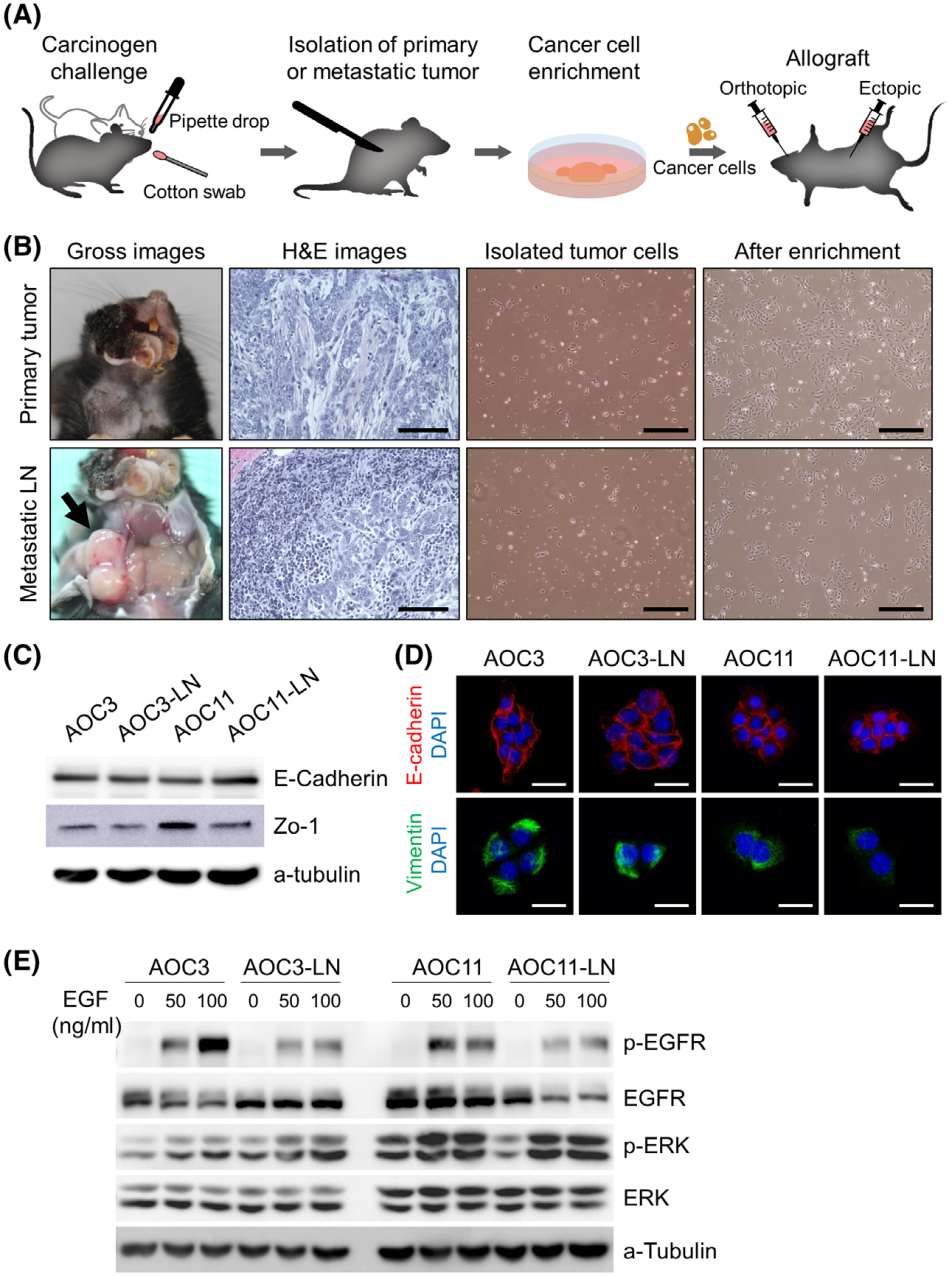
Isolation of carcinogen‐induced HNSCC cells and their molecular characterization. (A) Schematic diagram depicting the methods of establishing the murine syngeneic HNSCC model. (B) Representatives of the gross images of the carcinogen‐induced oral cancer (left panel), H&E stained images from the isolated tumors (left middle panel), light microscopic images of the isolated cells (right middle panel), and light microscopic images after enrichment with cell culture (right panel) from the primary tumor and metastatic lymph nodes, respectively. Scale bars, 100 μm in H&E images, 500 μm in right‐hand panels. Arrow in the lower left panel, metastatic lymph node (*n* = 17). (C) Expression of the epithelial markers, E‐cadherin and Zo‐1 in the established cell lines (*n* = 3). (D) Immunofluorescence images showing the membranous expression of E‐cadherin and the cytoplasmic expression of vimentin in the oral cancer cell lines. Scale bars, 50 μm (*n* = 3). (E) Western blot analysis showing p‐EGFR, EGFR, p‐ERK, and ERK protein levels in serum‐starved AOC3, AOC3‐LN, AOC11, and AOC11‐LN cells treated with 50 or 100 ng·mL^−1^ of epidermal growth factor (EGF) for 10 min. α‐Tubulin was used as the loading control (*n* = 3). H&E, hematoxylin and eosin; LN, lymph node; HNSCC, head and neck squamous cell carcinoma.

We developed a syngeneic tumor model by isolating representative murine HNSCC cells from DMBA‐induced oral cancers in C57Bl/6 mice mimicking human HNSCC carcinogenesis (Fig. 1A). Seventeen of the twenty mice administered DMBA developed tumors in the oral cavity (Fig. 1B). Two mice showed grossly overt metastatic tumors in the draining cervical LNs. Hematoxylin and eosin‐stained images of DMBA‐induced tumors showed histopathological characteristics similar to those of squamous cell carcinoma (SCC). We isolated cancer cells from primary (designated them AOC) and from metastatic LN tumors (designated as LNC)—19 HNSCC cell lines were established (Table 1). Next, the isolated cancer cell lines were transplanted into other mice to evaluate their tumorigenicity. Only four tumor cell lines (two AOCs and two LNCs) developed SCC when transplanted into C57Bl/6 mice (Fig. S1, Table 1). Furthermore, we showed that repeated painting with a cotton swab induced more stable tumor formation in the buccal mucosal area than the pipette drop method of carcinogen application.

**Table 1:** Information of the established cell lines regarding periods and methods for carcinogen challenge, and tumorigenicity. AOC, Ajou Oral Cancer; LNC, designated for established cancer cells from metastatic lymph nodes.

Designation	Periods for carcinogen challenge	Methods for carcinogen challenge	Tumorigenicity upon syngeneic inoculation
AOC1	27 weeks	Pipette drop	No
AOC2	27 weeks	Pipette drop	No
AOC3	28 weeks	Cotton swab	Yes
AOC3‐LN	28 weeks	Cotton swab	Yes
AOC4	29 weeks	Pipette drop	No
AOC5	29 weeks	Pipette drop	No
AOC6	29 weeks	Pipette drop	No
AOC7	31 weeks	Pipette drop	No
AOC8	31 weeks	Pipette drop	No
AOC9	31 weeks	Pipette drop	No
AOC10	31 weeks	Pipette drop	No
AOC11	31 weeks	Cotton swab	Yes
AOC11‐LN	31 weeks	Cotton swab	Yes
AOC12	31 weeks	Cotton swab	No
AOC13	33 weeks	Cotton swab	No
AOC14	33 weeks	Cotton swab	No
AOC15	35 weeks	Cotton swab	No
AOC16	35 weeks	Cotton swab	No
AOC17	35 weeks	Cotton swab	No

### Established murine HNSCC cells shared intrinsic cell properties and conserved mutational profile with human HNSCC

To determine whether the isolated murine HNSCC cells exhibited typical characteristics of human HNSCC, the expression of epithelial cell markers and key growth factor receptor signaling was evaluated. Western blotting revealed expression of the epithelial markers, E‐cadherin and Zo‐1, in the isolated AOC cells (Fig. 1C). Immunofluorescence images showed that the expression of E‐cadherin was well localized to the cytoplasmic membrane, while that of the mesenchymal marker, vimentin, was localized to the cytoplasm (Fig. 1D). Furthermore, stimulation with epidermal growth factor (EGF) increased the p‐EGF receptor and downstream p‐ERK expression in a dose‐dependent manner in all AOC cells. These results indicated that the isolated AOC cells exhibited intrinsic cell properties, including EGF responsiveness, similar to human HNSCC (Fig. 1E).

**Fig. 2:**
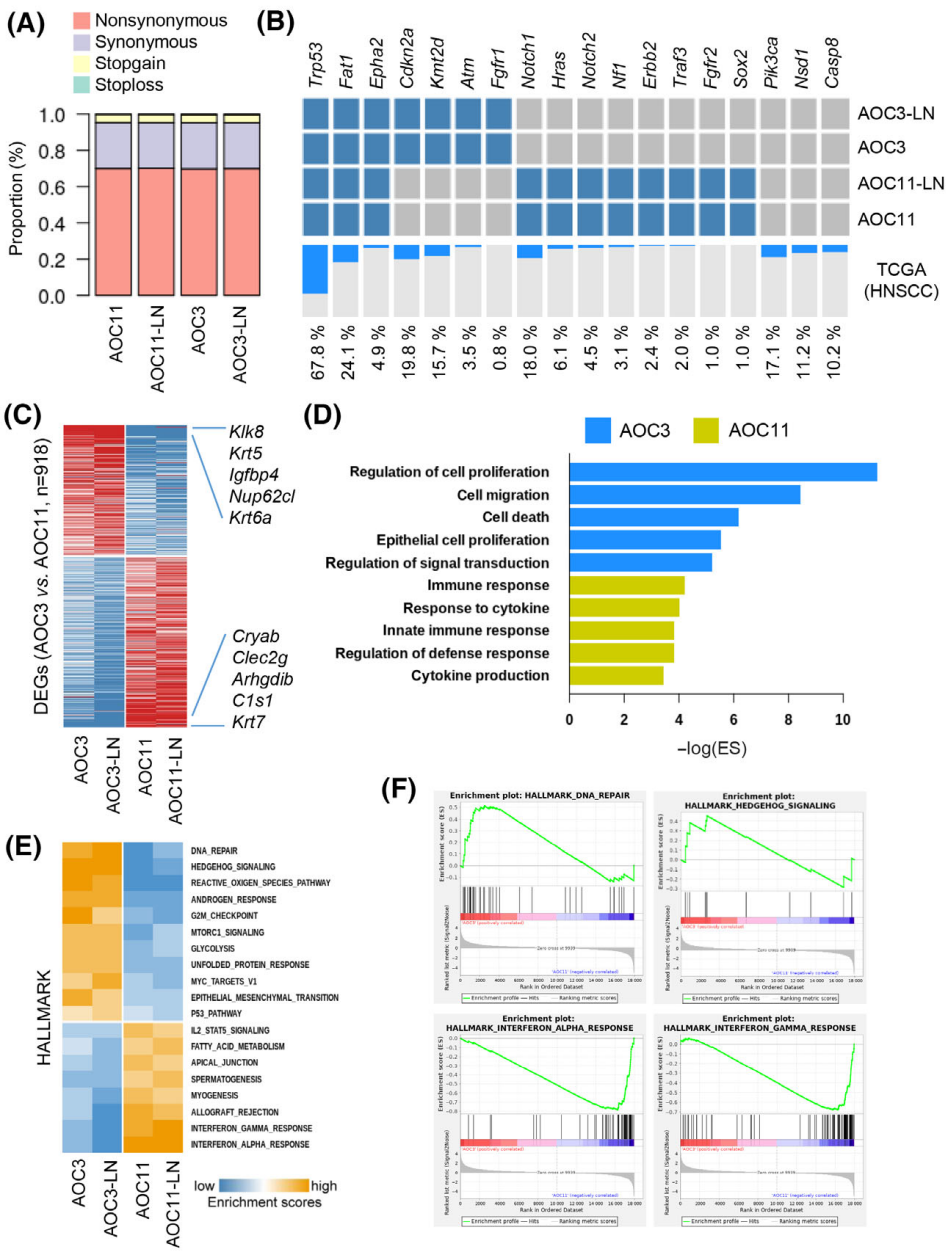
Whole exome sequencing and transcriptome analysis of the established HNSCC cell line. (A) Distribution of the mutation types according to protein functions in each sample (*n* = 3). (B) A heatmap shows the mutation profile of the established cell line for the previously reported frequently mutated genes (*n* = 18). A bar plot indicates the mutation frequencies of TCGA‐HNSCC data. (C) The heatmap shows the differentially regulated genes between the AOC3 and AOC11 cell lines. The top 5‐ranked genes with the greatest fold differences are indicated. (D) The bar plot shows the functional enrichment scores of DEGs in AOC3 (*n* = 398, blue) and AOC11 cells (*n* = 250, green). (E) The heatmap represents the AOC3‐ and AOC11‐specific enrichment scores for the biological pathways (MsigDB HALLMAKR) in each sample. (F) Gene set enrichment analysis shows the enriched expression of the DNA repair and Hedgehog signaling‐related genes in AOC3 cells compared to those in AOC11 cells (*upper*). It shows the enriched expression of the Interferon alpha and gamma response‐related genes in AOC11 cells compared to those in AOC3 cells (*lower*). DEG, differentially expressed gene; HNSCC, head and neck squamous cell carcinoma; TCGA, The Cancer Genome Atlas.

The mutational profiles of individual cell lines determined using next‐generation sequencing identified abundant non‐synonymous mutations (69.76–70.14%) in the four AOC cells (Fig. 2A). Furthermore, the comparison of the mutational profiles of the individual AOC lines with frequently mutated genes in human HNSCC (based on The Cancer Genome Atlas analysis [20]) revealed non‐synonymous mutations in *Trp53*, the most frequently mutated gene in human HNSCC tumorigenesis, in all four AOC cells (Fig. 2B). Other important genes, such as *Fat1*, were also mutated in AOC cell lines. Evaluation of HPV status showed that both AOC3 and AOC11 cells are HPV‐negative HNSCC. Enrichment scores of the HNSCC subtypes showed that all four cell lines are similar to the classical subtype of HNSCC rather that mesenchymal or atypical subtypes (Fig. S2A). These results indicated that the isolated murine AOC cells had mutational features similar to human HNSCC.

### Individual AOC cell lines showed distinct mutational profiles and molecular characteristics

Detailed evaluation of the mutational profile in individual AOC cell lines revealed distinct patterns of mutations. Although *Trp53*, *Fat1*, and *Epha2* mutations were detected in all cell lines, other important genes, including *Cdkn2a*, *Kmt2d*, *Notch1*, and *Hras*, were differentially mutated in AOC3 and AOC11 cells (Fig. 2B, Table S1). Moreover, the mutational patterns of two cell lines derived from LN‐metastatic tumors (AOC3‐LN and AOC11‐LN) were similar to those of respective AOCs (AOC3 and AOC11). Consistently, Jaccard similarity score according to the mutational profile showed the distinct characteristics between the AOC3 and AOC11 cells while the cells from the LN‐metastatic tumors (AOC3‐LN, AOC11‐LN) were similar with their parent cells (Fig. S2B). Although the same carcinogen challenge elicits common characteristics of mucosal SCC, such as EGF responsiveness and *Trp53* mutation, these results indicated that HNSCC cells isolated from different individual mice have distinct molecular profiles.

Next, we performed transcriptome analysis of AOC cells. The heat map revealed the global differences between ACO3 and AOC11 cells (Fig. S3A), and the comparison between these two cell lines identified 918 differentially expressed genes (DEGs) (fold change > 1; Fig. 2C, Table S2). The upregulated genes in AOC3 cells were involved in cell proliferation and migration, whereas those in AOC11 cells were responsible for the immune response and cytokine production (Fig. 2D). GSEA of the biological pathways revealed that the DNA repair, Hedgehog signaling, and reactive oxygen species pathways were upregulated in AOC3 cells (Fig. 2E). In contrast, the IFN response, allograft rejection, and IL‐2 signaling pathways were upregulated in AOC11 cells (Fig. 2F). These results suggest that AOC3 cells are more tumorigenic than AOC11 cells, which are related to antitumor immune responses. Consistent with these observations, orthotopic inoculation of the four cell lines resulted in more rapid tumor growth when injected with AOC3 and AOC3‐LN cells than when injected with AOC11 and AOC11‐LN cells (Fig. S4). Consistently, *in vitro* assays also showed higher abilities of cell viability as well as cell migration in AOC3 and AOC3‐LN cells when compared with those in AOC11 and AOC11‐LN cells (Fig. S5).

Further evaluation of the differences in gene expression profiles of AOC and AOC‐LN cell lines revealed similar expression patterns in most AOC‐LN and AOC cells derived from the same individual mice (Fig. S2B,C). However, detailed transcriptome analysis revealed a subset of DEGs and upregulated pathways in AOC‐LN cells compared with those in AOC cells (Fig. S2B–F).

### Syngeneic model with the established AOC cell line showed immunogenic TME and response to anti‐PD‐1 immunotherapy

**Fig. 3:**
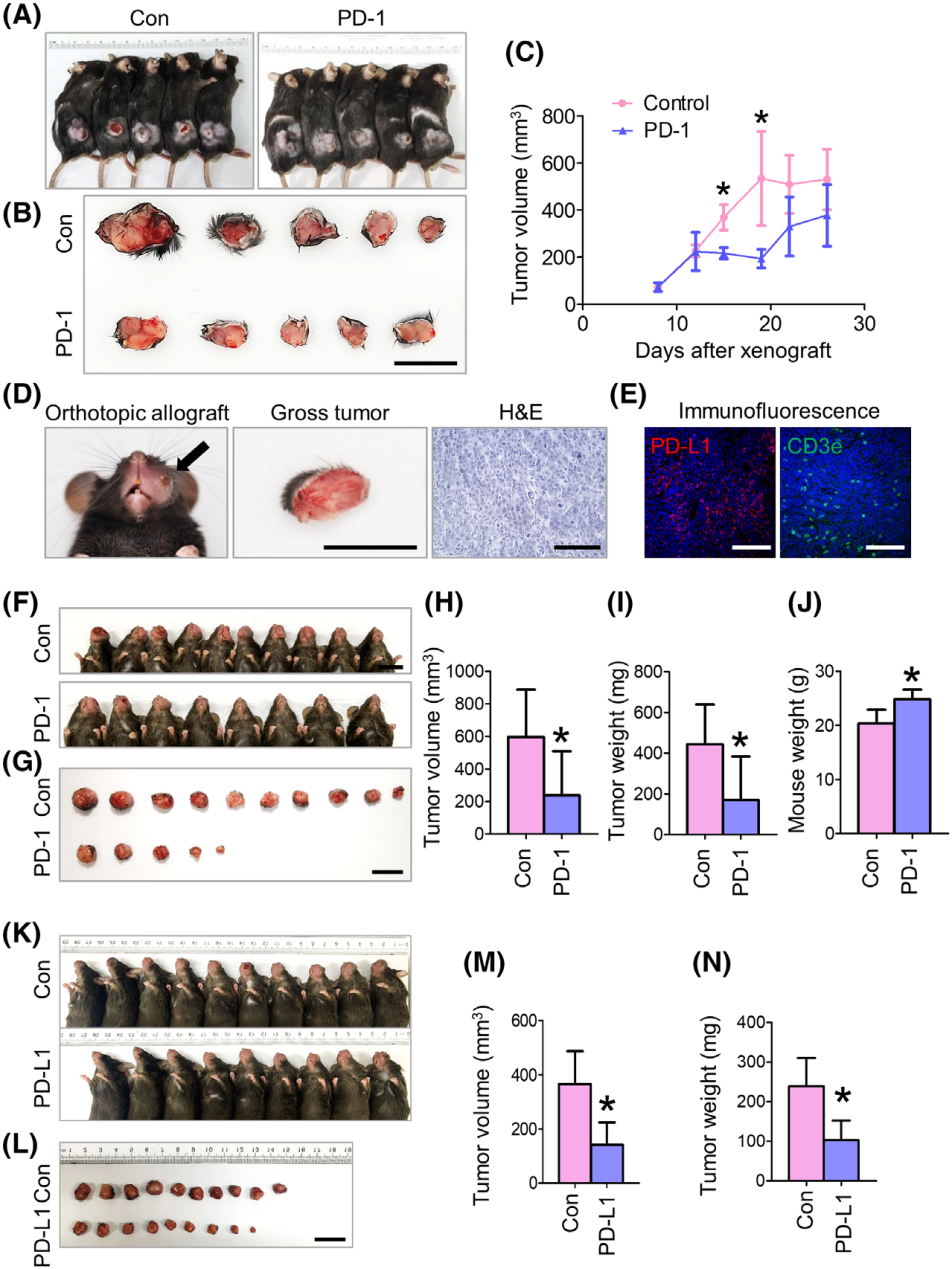
Established syngeneic orthotopic model shows reliable tumorigenicity, immunogenic tumor microenvironment, and anti‐PD‐1 or ‐PD‐L1 immunotherapy responses. (A, B) Gross images showing ectopic syngeneic tumors. Mice were subcutaneously injected with AOC3‐LN cells in the left flank. Scale bar, 20 mm. (C) Tumor growth curves of anti‐PD‐1 inhibitor‐treated and control groups. *n* = 5 in each group. Student's *T* tests were performed between groups and *P < 0.05 versus control. Data were presented mean ± SD. (D) Images showing the syngeneic tumor in the buccal area (left panel), gross tumor appearance (middle panel), and pathologic squamous cell carcinoma after H&E staining (right panel). Arrow indicates orthotopic oral cancer. Scale bars: 10 mm, middle panel; 500 μm, right panel. (E) Immunofluorescence images showing CD31: representing blood vessels, F4/80: representing macrophages, and CD3e: representing T‐cells in the tumor microenvironment. Scale bars, 100 μm. (F, G) Representative images showing orthotopic syngeneic tumors obtained from two repetitive experiment. Mice were submucosally injected with AOC3‐LN cells in the left buccal area. Con, control group; PD‐1, anti‐PD‐1 inhibitor‐treated group. Scale bars, 20 mm. (H–J) Comparisons of tumor volume, tumor weight, and mouse weight between control and anti‐PD‐1‐treated groups; Values are expressed as means ± SD, *n* = 10 in Control, *n* = 10 in PD‐1; Student's *T* tests were performed between groups and *P < 0.05 versus control. Representative results obtained from two repetitive experiments with the same number of mice, *n* = 10 in Control, *n* = 10 in PD‐1. (K, L) Representative images showing orthotopic syngeneic tumors obtained from two repetitive experiment. Mice were submucosally injected with AOC3‐LN cells in the left buccal area. Con, control group; PD‐L1, anti‐PD‐L1 inhibitor‐treated group. Scale bar, 20 mm. (M, N) Comparisons of tumor volume and weight between control and anti‐PD‐L1‐treated groups; Values are expressed as means ± SD, *n* = 10 in Control, *n* = 10 in PD‐L1; Student's *T* tests were performed between groups and *P < 0.05 versus control. Representative results obtained from two repetitive experiments with the same number of mice, *n* = 10 in Control, *n* = 10 in PD‐L1. H&E, hematoxylin and eosin; LN, lymph node; PD, programmed death.

AOC3‐LN cells showed the most reliable experimental tumorigenicity among the four cell lines (Fig. S3). Therefore, we performed *in vivo* experiments using AOC3‐LN cells to establish a reliable syngeneic model. Intraperitoneal injection of anti‐PD‐1 inhibitors resulted in a delayed tumor growth curve in the ectopic syngeneic model (Fig. 3A–C). Subsequently, we established an orthotopic syngeneic model to represent a more reliable TME, especially for oral cancers. Orthotopic buccal injection of AOC3‐LN resulted in reliable tumor growth and histopathologically indicated SCC (Fig. 3D). Immunofluorescence imaging of the TME showed the expression of PD‐L1 and infiltration of CD3^+^ T‐cells in the intratumoral area, indicating that the AOC3‐LN syngeneic tumors were PD‐L1‐expressing and T‐cell‐infiltrated type (Fig. 3E).

Subsequent administration of anti‐PD‐1 inhibitors to the orthotopic buccal syngeneic model decreased the tumor volume and weight in the treatment group compared to those in the control group (Fig. 3F–J). Three of the eight mice in the anti‐PD‐1 inhibitor group showed no evidence of oral cancer 3 weeks after cancer cell injection. In contrast, all mice in the control group showed macroscopic oral cancers (Fig. 3F,G), suggesting tumor regression after immunotherapy occurs in a subset of mice. Furthermore, the body weights of the mice 3 weeks after orthotopic tumor cell injection were significantly increased in the anti‐PD‐1 inhibitor group compared with those in the control group (Fig. 3J). Treatment with anti‐PD‐L1 inhibitors also resulted in decreased tumor volume and weight in the orthotopic syngeneic model (Fig. 3K–N). These results demonstrated that the syngeneic model established here exhibited an immunogenic TME and responded to anti‐PD‐1 immunotherapy.

### ICB treatment elicits increased intratumoral T‐cell infiltration and decreased blood vascular density with improved pericyte coverage

**Fig. 4:**
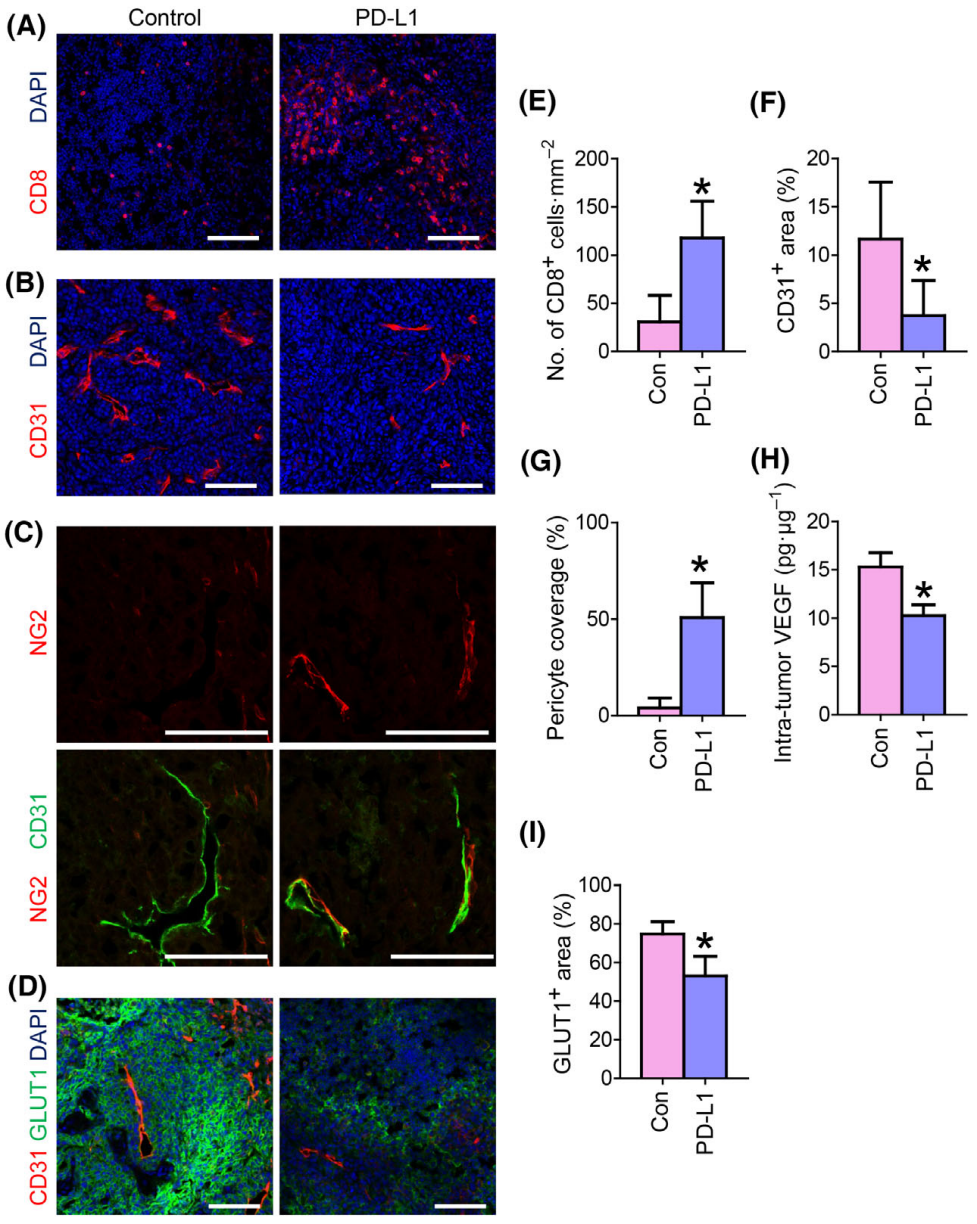
Anti‐PD‐L1 treatment elicits increased intramucosal T‐cell infiltration and decreased blood vascular density with proper pericyte coverage. Mice were submucosally injected with 5 × 10^5^ AOC3‐LN cells in the left buccal area and intraperitoneally injected with an anti‐PD‐L1 inhibitor (10 mg·kg^−1^) twice a week for 3 weeks. (A–D) Immunofluorescence images showing CD8: representing T‐cells, CD31: representing blood vessels, NG2: representing pericyte coverages, and GLUT1: representing hypoxic condition expression level in the tumor microenvironment. Scale bars, 100 μm. (E–H) Comparisons of CD8: representing T‐cells, CD31: representing blood vessels, NG2: representing pericyte coverages, and GLUT1: representing hypoxic condition expression level between control and anti‐PD‐1‐treated groups; Values are expressed as means ± SD, *n* = 6 in Control, *n* = 6 in PD‐L1; Student's *T* tests were performed between groups and *P < 0.05 versus control. (I) Intratumor VEGF levels were assessed using an ELISA kit; Values are expressed as means ± SD, *n* = 6 in Control, *n* = 6 in PD‐L1; Student's *T* tests were performed between groups and *P < 0.05 versus control. Representative results obtained from three repetitive experiments with the same number of mice. ELISA, enzyme‐linked immunosorbent assay; LN, lymph node; PD, programmed death; VEGF, vascular endothelial growth factor receptor.

Next, we evaluated tumor microenvironmental changes in HNSCC after ICB therapy using immunofluorescence imaging. Treatment with the anti‐PD‐L1 inhibitor showed a marked increase in CD8^+^ and CD4^+^ T‐cell infiltration inside the tumor, indicating an immunogenic microenvironment (Fig. 4A,E, Fig. S6A,D). On the other hand, myeloid cells including F4/80^+^ macrophages and Ly6G^+^ neutrophils were slightly decreased or not changed after anti‐PD‐L1 treatment (Fig. S6B,C,E,F). Importantly, immunostaining for CD31, a blood vessel marker, showed a significant decrease in blood vessel density after anti‐PD‐L1 treatment (Fig. 4B,F). Immunostaining for NG2, a pericyte marker, revealed increased NG2^+^ pericyte coverage in CD31^+^ vascular endothelial cells (Fig. 4C,G). In naïve HNSCC tumors, blood vessels showed chaotic morphology with multiple sprouting phenomena. Anti‐PD‐L1 treatment decreased vascular densities and sprouting, indicating the normalization of the vasculature after ICB treatment. In line with these observations, intratumoral VEGF‐A levels were significantly decreased after anti‐PD‐L1 treatment (Fig. 4H). The immunofluorescence staining of GLUT1 showed decreased expression in PD‐L1 inhibitor‐treated tumors compared to that in control tumors, indicating that tumor hypoxia was improved after ICB treatment (Fig. 4D,I).

### Anti‐PD‐L1‐induced normalization of TME in HNSCC is modulated via type I IFN pathway

**Fig. 5:**
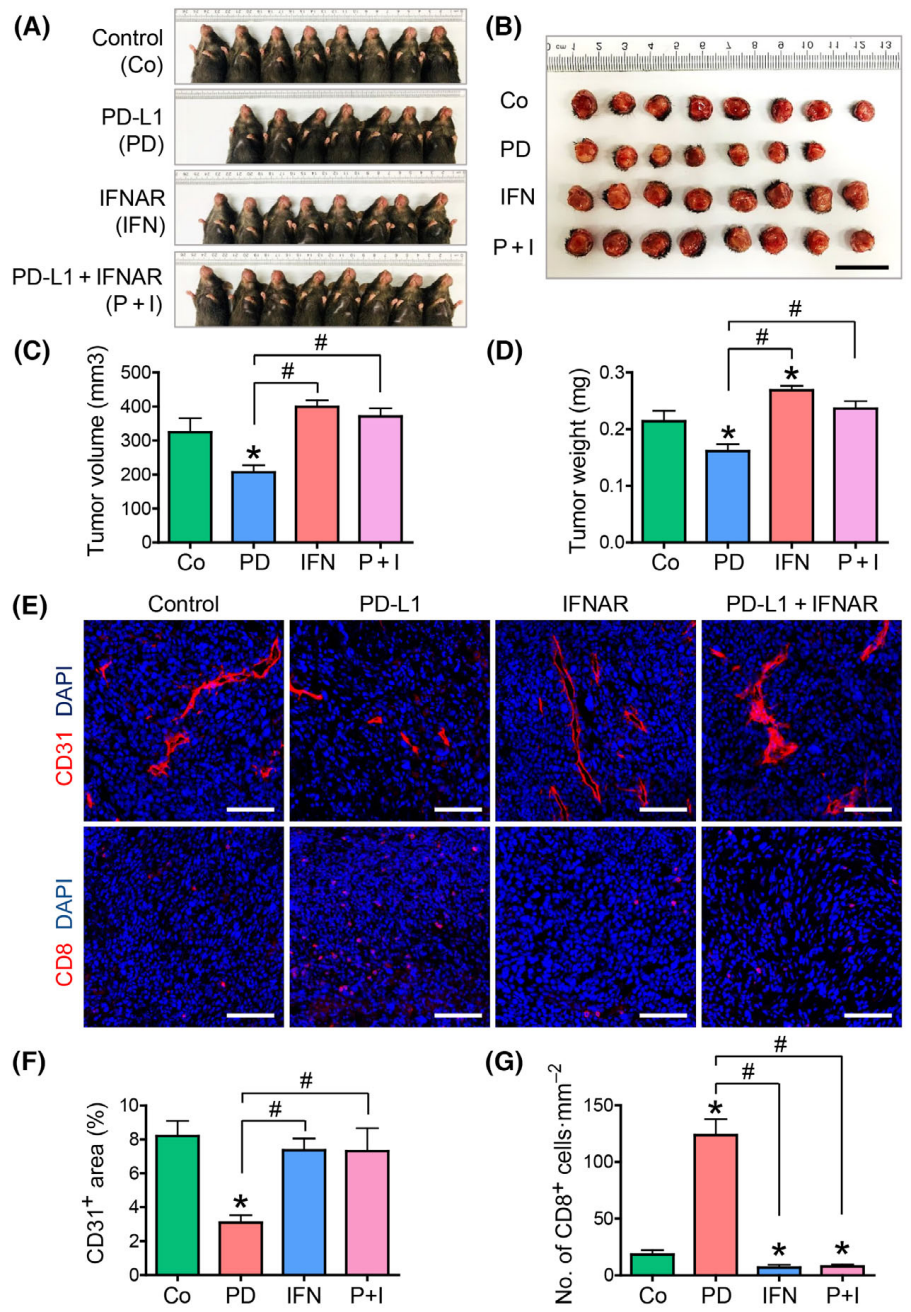
Treatment with IFNAR abrogates the anti‐PD‐L1 treatment‐induced vascular changes. (A, B) Gross images showing orthotopic syngeneic tumors. Mice were submucosally injected with AOC3‐LN cells in the left buccal area. Co, control group; PD, anti‐PD‐1 inhibitor‐treated group; IFN, anti‐IFNAR inhibitor‐treated group; P + I, anti‐PD‐1 and anti‐IFNAR treated group; *n* = 8 in Control, *n* = 7 in PD‐L1, *n* = 8 in IFNAR, *n* = 8 in PD‐L1 + IFNAR. Scale bar, 20 mm. (C, D) Comparisons of tumor volume and weight in mice; Values are expressed as means ± SD, *n* = 8 in Control, *n* = 7 in PD‐L1, *n* = 8 in IFNAR, *n* = 8 in PD‐L1 + IFNAR; *P < 0.05 versus control; ^#^ *P* < 0.05 versus anti‐PD‐L1. Representative images (E) and comparisons (F, G) of CD31: representing blood vessels and CD8: representing T‐cells in tumors. Values are expressed as means ± SD, *n* = 8 in Control, *n* = 7 in PD‐L1, *n* = 8 in IFNAR, *n* = 8 in PD‐L1 + IFNAR; Scale bar, 100 μm. Student's *T* tests were performed between groups and *P < 0.05 versus Control: ^#^ *P* < 0.05 versus anti‐PD‐L1. Representative results obtained from three repetitive experiments with the same number of mice. IFNAR, anti‐interferon alpha/beta receptor; LN, lymph node; PD, programmed death.

Considering that the PD‐1/PD‐L1 axis in the tumor is mainly associated with the interaction between cancer and immune cells, blood vascular changes after ICB treatment are thought to have indirect effects on the TME. Several studies have shown the immune‐vascular interaction and the pivotal role of Type I IFN signaling; however, its significance in HNSCC is yet to be evaluated [19, 21]. Here, we evaluated the tumors by administering an anti‐PD‐L1 ICB combined with an anti‐IFNAR inhibitor in a syngeneic model. Intraperitoneal injection of anti‐PD‐L1 inhibitors delayed tumor growth. Moreover, the anti‐PD‐L1‐induced reductions in tumor volume and weight were completely abrogated by combined treatment with an anti‐IFNAR inhibitor, indicating that Type I IFN signaling is indispensable for ICB‐induced antitumor effects in HNSCC (Fig. 5A–D). Furthermore, immunofluorescence imaging revealed that combined treatment with anti‐IFNAR therapy alleviated the anti‐PD‐L1 inhibitor‐induced tumor vessel normalization (Fig. 5E,F), indicating the restoration of the anti‐PD‐L1 inhibitor‐induced alterations in TME by an anti‐IFNAR inhibitor. After Type I IFN blockade, the tumor vessels showed increased density and chaotic morphology with increased endothelial sprouting (Fig. 5E). The increase in CD8^+^ T‐cell infiltration after ICB treatment was also abolished by the combined administration of an anti‐IFNAR inhibitor (Fig. 5E,G). These findings indicate that the anti‐PD‐L1‐induced antitumor effect and TME normalization depend on Type I IFN signaling.

### Comparison of the tumor microenvironmental changes between cisplatin, anti‐VEGFR2, and anti‐PD‐L1 treatments

**Fig. 6:**
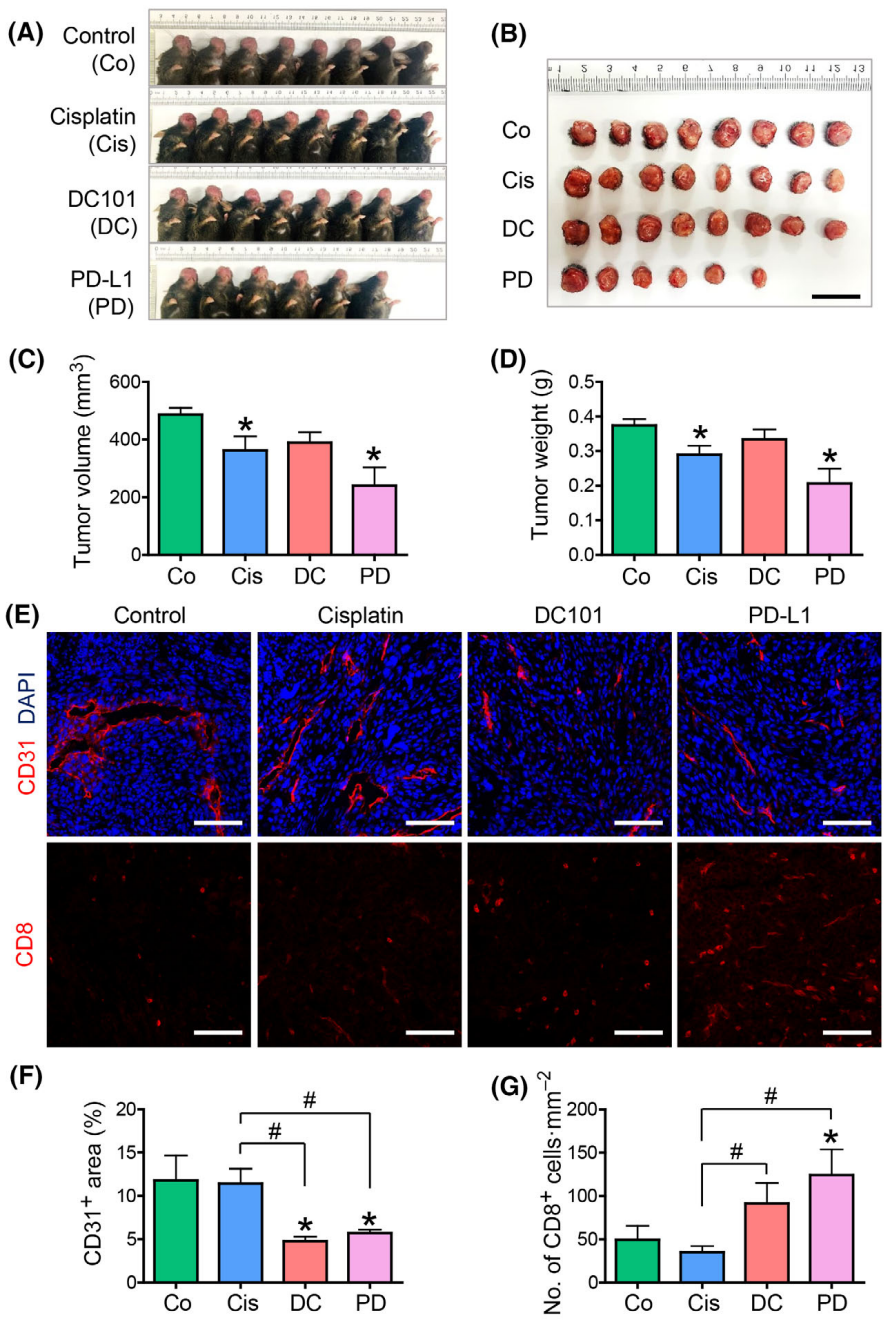
Comparisons of the tumor microenvironmental normalization between cisplatin, anti‐VEGFR2, and anti‐PD‐L1 treatments. (A, B) Gross images showing orthotopic syngeneic tumors. Mice were submucosally injected with AOC3‐LN cells in the left buccal area. Co, control group; Cis, cisplatin‐treated group; DC, DC101 (anti‐VEGFR‐2) treated group; PD, anti‐PD‐L1 treated group. *n* = 8 in Control, *n* = 8 in cisplatin, *n* = 8 in DC101, *n* = 6 in PD‐L1. Scale bar, 20 mm. (C, D) Comparisons of tumor volume and weight in mice. Values are expressed as means ± SD, *n* = 8 in Control, *n* = 8 in cisplatin, *n* = 8 in DC101, *n* = 6 in PD‐L1; Student's *T* tests were performed between groups and *P < 0.05 versus control. Representative images (E) and comparisons (F, G) of CD31; representing blood vessels and CD8: representing T‐cells in the tumor. Values are expressed as means ± SD, *n* = 8 in Control, *n* = 8 in cisplatin, *n* = 8 in DC101, *n* = 6 in PD‐L1; Scale bar, 100 μm. Student's *T* tests were performed between groups and *P < 0.05 versus control. ^#^ *P* < 0.05 versus anti‐PD‐L1. Representative results obtained from three repetitive experiments with the same number of mice. anti‐VEGFR‐2, anti‐vascular endothelial growth factor receptor 2; LN, lymph node; PD, programmed death.

Next, we compared the antitumor effects of anti‐PD‐L1 treatment with various other cancer therapeutics, including cisplatin, one of the most widely used chemotherapeutics, and DC101, a potent anti‐angiogenic agent, by VEGFR2 blockade. Treatment with cisplatin (5 mg·kg^−1^) and anti‐PD‐L1 inhibitors significantly reduced tumor size and weight, whereas VEGFR2 blockade did not (Fig. 6A–D). Furthermore, the immunofluorescence visualization of the TME revealed no significant changes in tumor vessels or CD8^+^ T‐cell infiltration after cisplatin treatment (Fig. 6E–G). These results suggest that the antitumor effects of cisplatin mainly occur via direct tumor cell killing, whereas its effects on stromal cells are limited. The administration of DC101 resulted in decreased vascular density and a modest increase in CD8^+^ T‐cell infiltration. However, the anti‐VEGFR2‐induced changes in stromal cells were not associated with delayed tumor growth. Administration of an anti‐PD‐L1 inhibitor resulted in decreased vascular densities and increased CD8^+^ T‐cell infiltration in TMEs (Fig. 6E–G), and these effects were combined with potent antitumor effects (Fig. 6A–D). These results indicate that in PD‐L1‐expressing HNSCC, treatment with ICB resulted in tumor microenvironmental normalization and subsequent antitumor effects.

## Discussion

As tumor immunotherapy is emerging as a next‐generation therapy to treat solid cancers, the unmet need for translational research is increasing [6]. Appropriate preclinical models are prerequisites for immunotherapy research in many individual laboratories [14]. Here, we refined the methods generating a murine syngeneic model of HNSCC to study cancer immunotherapy and reported the serial steps and key findings, which involved repeated carcinogen application in 20 mice, isolation of tumor cells, molecular and genetic characterization, the establishment of an *in vivo* syngeneic model, validation of the TME, and responses to immunotherapy. Through a comprehensive analysis of the genetic and molecular characteristics and *in vivo* microenvironment, our model system was proven to mimic human HNSCC with PD‐L1 expression.

A preclinical model system is one of the most widely used research platforms to investigate the aforementioned biological phenomena. Several model systems have been proposed to study HNSCC immunotherapy, each with advantages and drawbacks [11, 13, 14, 22, 23]. Besides the syngeneic tumor model, other preclinical rodent models can potentially be utilized to study cancer immunotherapy [11]. In HNSCC, several transgenic mouse models that allow oncogene activation and/or tumor suppressor inactivation in the epithelia of the head and neck mucosa have provided some promising aspects, as they are expected to have a naïve TME [22, 24, 25, 26, 27]. However, low penetrance rates, long latencies, limited tumor mutational burden, and minimal genetic diversity limit their practical utility [11, 22]. A humanized mouse model comprising immunodeficient host mice, human immune cells, and human tumor cells or tissues has been developed to recapitulate the interactions between immune components and tumors of human origin [28, 29]. The humanized tumor model has the major advantage of utilizing the human immune system, making it one of the most desirable systems. However, several limitations restrict their applicability, including xenograft‐versus‐host disease, incomplete reconstitution of certain immune subpopulations, and practical hurdles of high cost and laborious work [28].

The syngeneic tumor cell line model is the most commonly used preclinical model for evaluating immunotherapies, regardless of the type of cancer, owing to its ease of use and experimental reproducibility [11]. Previous studies have reported the reliable establishment of syngeneic HNSCC models and made important progress in immuno‐oncology research [13, 14, 30]. However, most other studies lack a preclinical platform and related information regarding its generation—it appears that the research time and costs were too high to generate such a model, making researchers hesitant to start the project. In this study, with repeated carcinogen application in 20 mice, we reported the development of clinically relevant macroscopic oral cancers in > 80% (*n* = 17) of mice. Of the 19 tumor cells from 17 mice, four cancer cell lines were established as reliable syngeneic tumor models. It took approximately 1 year to culture the cell lines and did not incur high research costs. We speculate that these results will be helpful to other researchers in developing their preclinical platforms and accelerate the field of immuno‐oncology research on HNSCC.

Our AOC cells exhibited molecular characteristics such as epithelial marker expression, EGF responsiveness, pathological SCC, and the conserved genomic characteristics harboring similar rates of non‐synonymous mutations, including those in *Trp53*, resembling human HNSCC. Furthermore, the cell lines from different mice showed distinct genomic characteristics. These results suggest that the syngeneic tumor models obtained from different cell lines exhibit distinct tumor characteristics, although they largely share conserved characteristics with mucosal SCCs. A recent study reporting the genomic characterization of human HNSCC cell lines showed cell line‐dependent genomic heterogeneity [31]. Therefore, a precise evaluation of genomic and molecular characteristics should be performed for each murine cell line obtained from chemical carcinogen‐induced tumors.

Using an *in vivo* syngeneic model, we further investigated the effects of ICB on tumors and TME. Although recent clinical data (KEYNOTE‐048 trial) have proven that ICB monotherapy is superior in PD‐L1‐expressing HNSCC as first‐line therapy compared with conventional chemotherapies [8, 32, 33], relevant basic research is still lacking. In this study, a preclinical model combined with immunofluorescence imaging allowed the evaluation of ICB treatment‐induced changes in TME in HNSCC. We showed that treatment with ICB alone was sufficient to induce vascular normalization (decreased tumor blood vessel density, improved pericyte coverage, and decreased intratumor VEGF‐A levels), the subsequent rescue of tissue hypoxia, and increased intratumoral T‐cell infiltration (Fig. 4). Considering that the PD‐1/PD‐L1 axis is generally dominant in the interaction between tumor and immune cells, such as T‐cells, ICB treatment could influence the global TME, including tumor‐associated blood vessels, tissue hypoxia, and T‐cell infiltration. Although studies have shown the efficacy of immunotherapy in normalizing tumor blood vessels [21, 34, 35], immunotherapy‐mediated vascular changes and TME normalization have not been fully elucidated. Nevertheless, our model can assist in understanding immune‐vascular crosstalk in the HNSCC microenvironment.

Our results showed that the Type I IFN pathway is indispensable for mediating ICB‐induced antitumor effects and TME normalization. Type I IFNs (mostly IFN‐α and IFN‐β) binds to a dimeric transmembrane receptor, IFNAR, that is linked to two cytosolic tyrosine kinases (TYK2 and JAK1) and activates transcription factors of the STAT family [36]. They were first described for their strong antiviral properties, and mounting evidence has accumulated regarding their antitumor activities [37]. Type I IFNs have multiple functions in controlling immune cells and are known to be associated with immunovascular crosstalk in tumors [19, 37]. The blockade of tumor microenvironmental normalization by IFNAR (Fig. 5) indicates the critical role of the Type I IFN pathway during immunotherapy and regulation of TME. In fact, the idea that PD‐L1 is a biomarker of response to checkpoint blockade as a result of underlying IFN responses has already been argued [38]. However, the role of type I IFN pathway in HNSCC immunotherapy has not been fully elucidated as far as we know. Our data using HNSCC‐specific syngeneic model that showed the critical role of type I IFN signaling during ICB responses could contribute the current understanding of the HNSCC biology and provide mechanistic insights on how ICB monotherapy works. Further studies will be of interest to elucidate the reciprocal interactions among Type I IFNs and various cell types, including cancer, immune, and endothelial cells in TME.

## Conclusions

Here, we refined the methods generating an orthotopic syngeneic model of HNSCC with reliable tumorigenic potential, conserved genomic and molecular characteristics similar to those of human HNSCC, and an immunogenic TME. ICB monotherapy was sufficient to induce tumor microenvironmental normalization, including T‐cell infiltration and vascular normalization. The Type I IFN pathway may be a key mediator of the antitumor effects of ICB. Our results provide mechanistic insights into the KEYNOTE‐048 clinical trial on how ICB monotherapy works in PD‐L1^+^ HNSCC. The described methods and key findings may help other researchers expand their research on HNSCC immunotherapy.

## Conflict of interest

The authors declare no conflict of interest.

## Author contributions

Conception and design: JYJ, C‐HK; Development of methodology: JYJ, B‐SL, MH, CS; Acquisition of data (provided animals, provided facilities, etc.): JYJ, B‐SL, MH, CS, J‐HC; Analysis and interpretation of data (e.g., statistical analysis, biostatistics, computational analysis): JYJ, B‐SL, MH, CS, J‐HC, YSS, HGW; Writing, review, and/or revision of the manuscript: JYJ, B‐SL, MH, J‐HC, HGW, C‐HK; Administrative, technical, or material support (i.e., reporting or organizing data, constructing databases): JYJ, B‐SL, MH, J‐HC, HGW; Study supervision: C‐HK.

### Peer review

The peer review history for this article is available at https://www.webofscience.com/api/gateway/wos/peer‐review/10.1002/1878‐0261.13633.

## Supporting information


**Fig. S1.** Syngeneic inoculation of the established cell lines.
**Fig. S2.** Heatmap showing enrichments scores of the HNSCC subtypes and Jaccard similarity scores among mutational profiles of the established cell lines.
**Fig. S3.** Transcriptome profile of the established cell lines.
**Fig. S4.** Orthotopic syngeneic model.
**Fig. S5.** Cell viability and migration assays of the established cell lines.
**Fig. S6.** Changes of immune cell infiltration in syngeneic tumor model after anti‐PD‐L1 treatment.
**Table S1.** Mutational profiles of the established cell lines.

**Table S2.** List of differentially expressed genes between AOC3 and AOC11 cells.

## Data Availability

The raw and processed transcriptome data generated in this study have been deposited in the GEO database under accession code GSE248271 (https://www.ncbi.nlm.nih.gov/geo/query/acc.cgi?acc=GSE248271). The raw data of whole exome sequencing has bene deposited into the NCBI Bioproject database under the BioProject ID PRJNA1042869 (https://www.ncbi.nlm.nih.gov/sra/PRJNA1042869).
