# Supplementary material for: Immune checkpoint inhibitor monotherapy is sufficient to promote microenvironmental normalization via the type I interferon pathway in PD‐L1‐expressing head and neck cancer
Source: Mol Oncol. 2024 Mar 21;18(8):1923–39. doi: 10.1002/1878-0261.13633 (PMC11306519; doi:10.1002/1878-0261.13633)
Supplement: Supplementary file 1 — Fig. S1. Syngeneic inoculation of the established cell lines. Fig. S2. Heatmap showing enrichments scores of the HNSCC subtypes and Jaccard similarity scores among mutational profiles of the established cell lines. Fig. S3. Transcriptome profile of the established cell lines. Fig. S4. Orthotopic syngeneic model. Fig. S5. Cell viability and migration assays of the established cell lines. Fig. S6. Changes of immune cell infiltration in syngeneic tumor model after anti‐PD‐L1 treatment. Table S1. Mutational profiles of the established cell lines. [file MOL2-18-1923-s001.docx]

**Supplementary Figures**

**
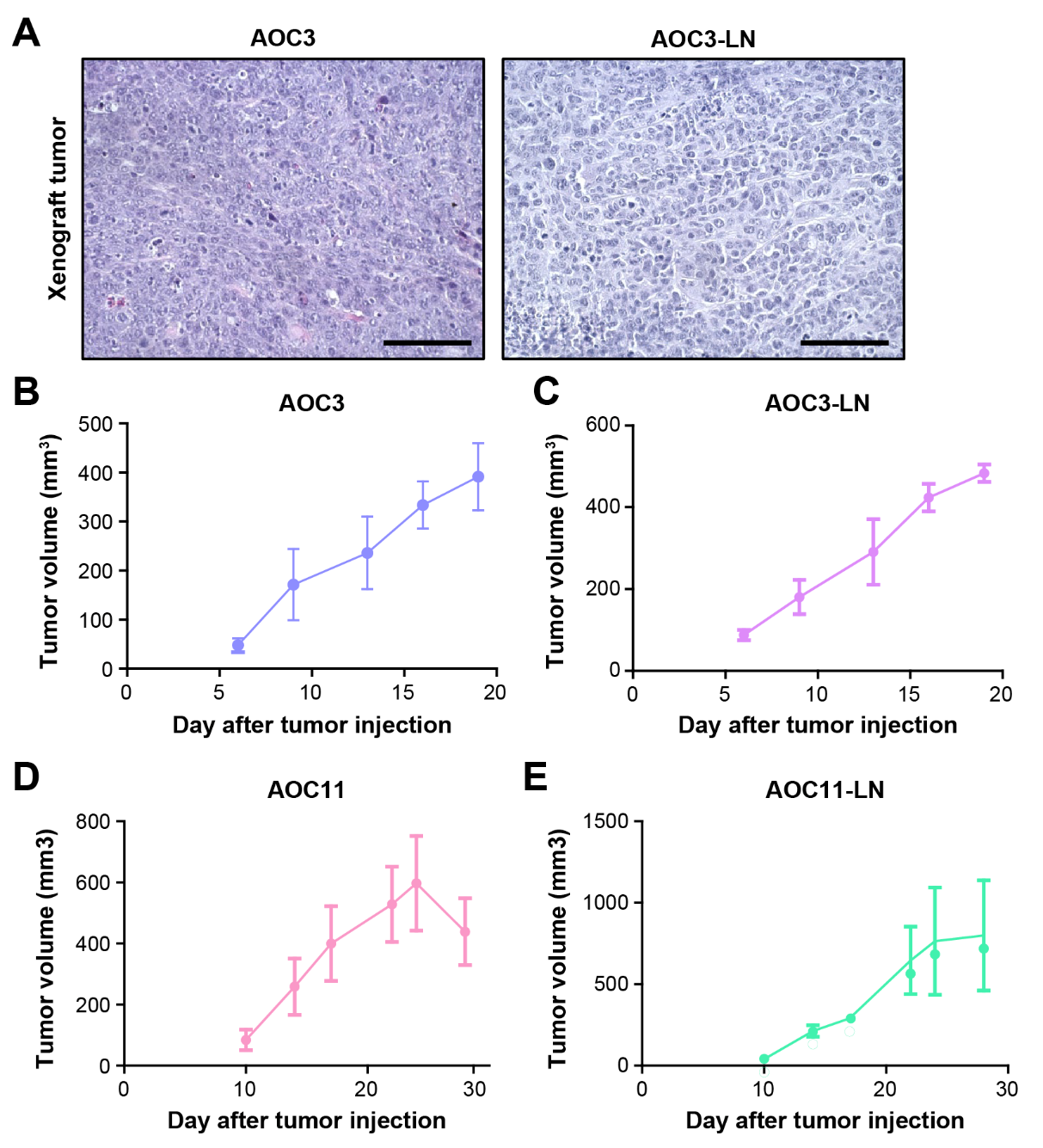
**

**Supplemental Figure S1** (A) H&E stained images of the syngeneic tumors showing pathological squamous cell carcinoma. To generate syngeneic tumor model, 5×10^5^ Ajou Oral Cancer (AOC) cells were injected subcutaneously into the flank area. The tumors were isolated 3 weeks after inoculation. Scale bars, 100 µm. (B–E) Tumor growth curve of the established syngeneic model using four oral cancer cell line.

**
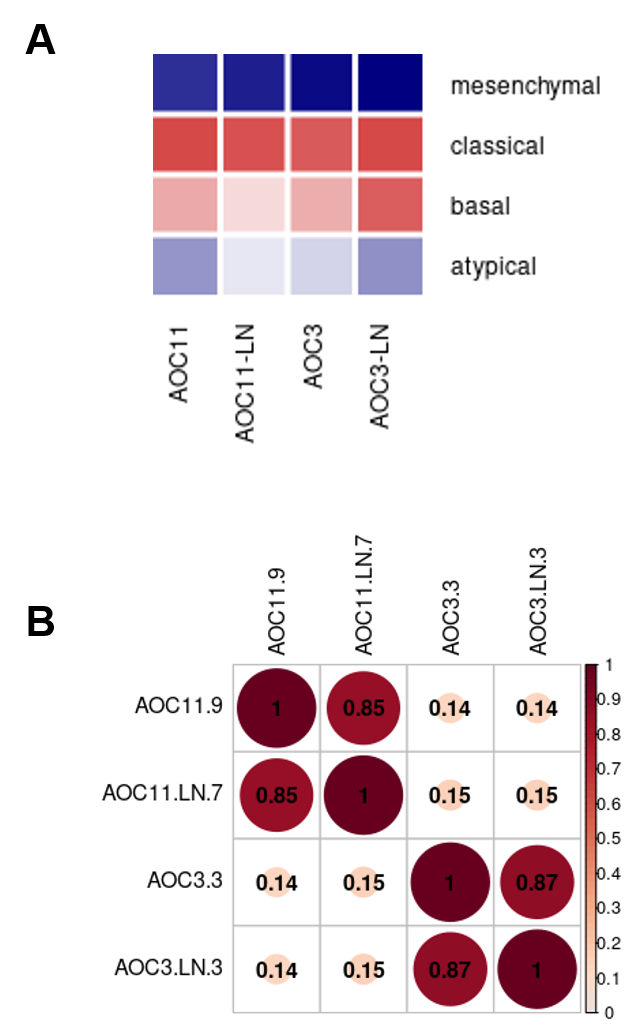
**

**Supplementary Figure S2** (A) A heatmap showing enrichment scores for the HNSCC subtypes across 4 cell lines. The enrichment score is estimated using the ssGSEA method. (B) Jaccard similarity scores among the mutation profiles of four cell lines.

**
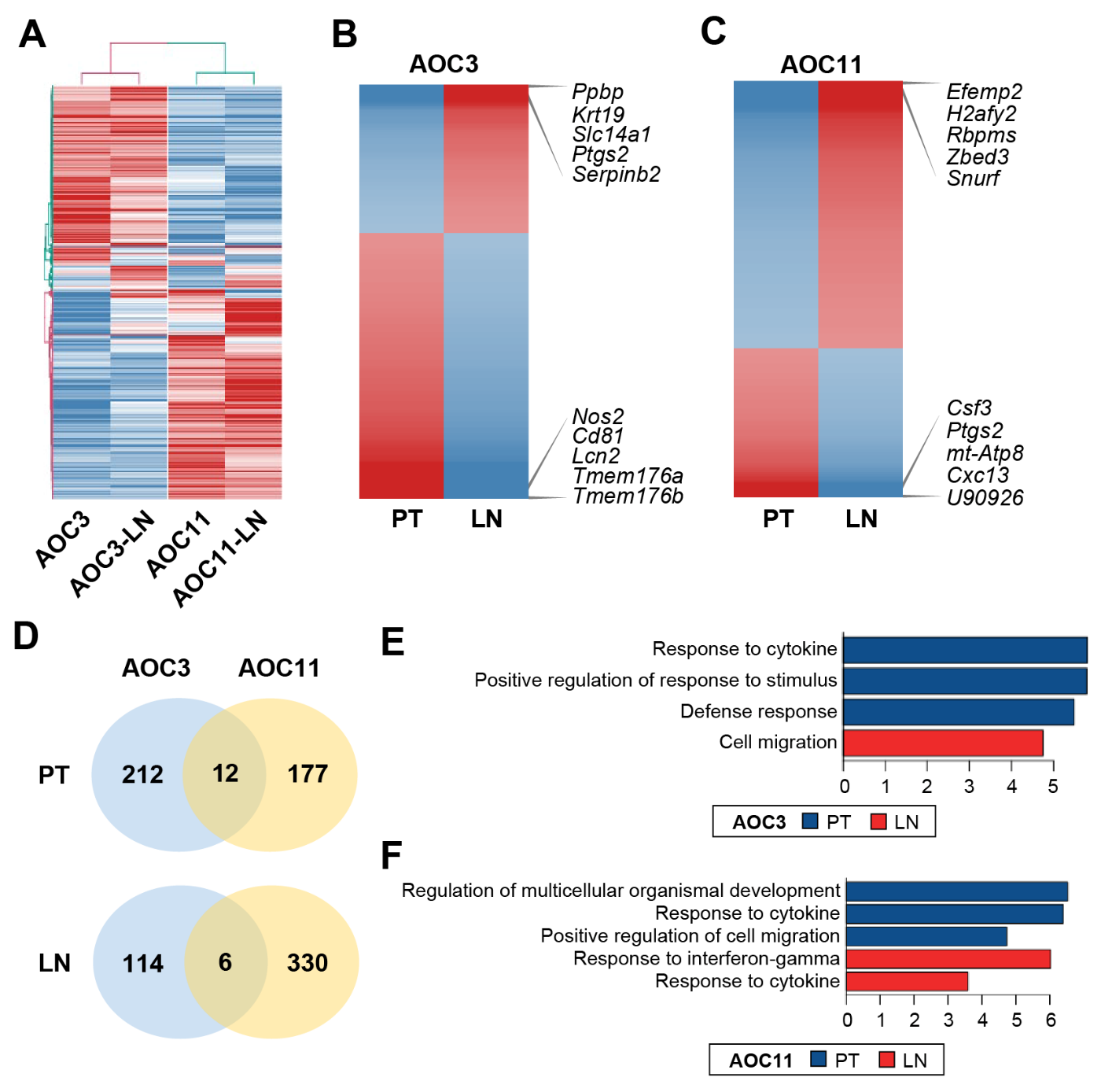
**

**Supplemental Figure S3** Transcriptome profile of the established cell line. (A) Unsupervised clustering of gene expression profiles of the established cell lines. (B,C) Differentially regulated genes between the primary tumor (PT) and lymph node (LN) in the AOC3 cells (B) and AOC11 cells (C) are shown. (D) Venn-diagrams show the number of up-regulated differentially expressed genes in PT (*upper*) and LN (*lower*) from each of AOC3 and AOC11. (E,F) Barplots show the functional enrichment scores for DEGs (PT *vs.* LN) in each AOC3 cell (E) and AOC11 cell (F).

**
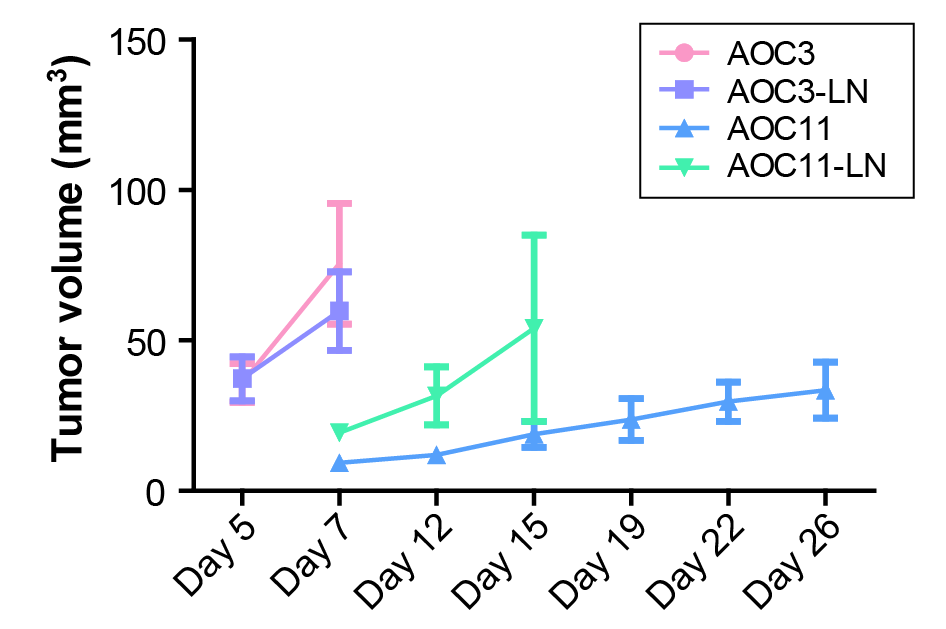
**

**Supplemental Figure S4** Tumor growth curves after orthotopic syngeneic inoculation of the four established cell line shows differential growth patterns of the individual cell line. For orthotopic model, a total of 2×10^5^ injected into the submucosal area of the oral tongue.


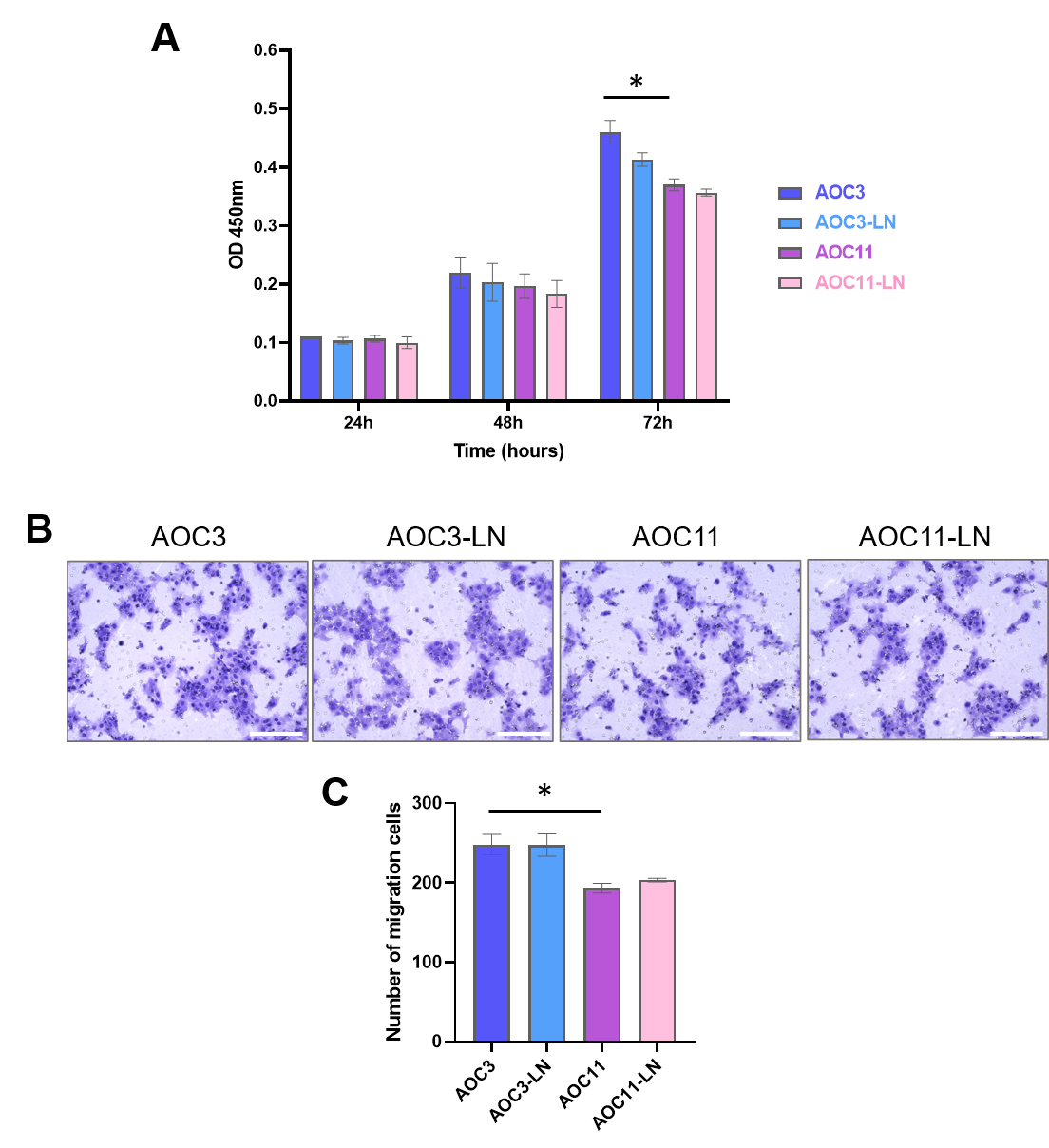


**Supplementary Figure S5** (A) Cell viabilities of AOC3, AOC3-LN, AOC11, AOC11-LN cells were measured by CCK8 for 24h, 48h and 72h. Values are expressed as means ± SD from three independent experiments (*P < 0.05). (B) the migrated cells were photographed with a microscope and counted at 24 h. Scale bars = 200um. (C) Quantification data. mean±SD from three independent experiments. *P < 0.05.


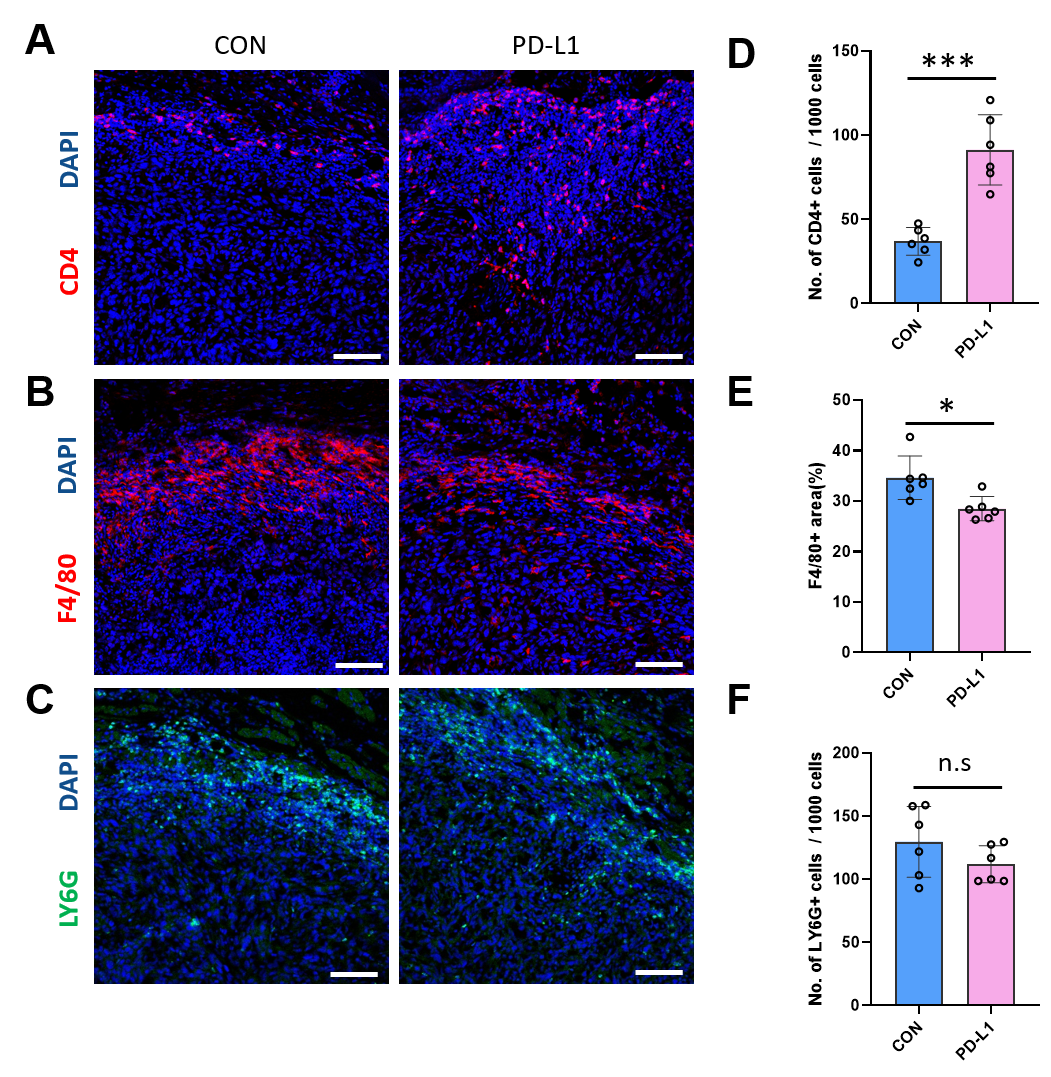


**Supplementary Figure S6** Mice were submucosally injected with 5 × 10^5^ AOC3-LN cells in the left buccal area and intraperitoneally injected with an anti-PD-L1 inhibitor (10 mg/kg) twice a week for 3 weeks. (A–C) Immunofluorescence images showing CD4^+^ T-cells, F4/80 macrophage, LYG6 neutrophil. Scale bars, 200 µm. (D–F) Comparisons of CD4^+^ T-cells, F4/80 macrophage, LYG6 neutrophil, n = 6; ^*^*P* < 0.05 , ^* * *^*P* < 0.001 versus control.

**Supplemental Table S1. Mutational profiles of the established oral cancer cell lines.**

| **Sample** | **Chr** | **Start** | **End** | **Ref** | **Alt** | **Func.knownGene** | **Gene.knownGene** | **ExonicFunc.knownGene** | **ReadDepth (MUT)** | **ReadDepth (WT)** | **VAF** |
| --- | --- | --- | --- | --- | --- | --- | --- | --- | --- | --- | --- |
| AOC11.9 | 11 | 69588505 | 69588505 | G | A | exonic | Trp53 | nonsynonymous SNV | 33 | 43 | 0.43 |
| AOC11.9 | 11 | 69588676 | 69588676 | G | T | exonic | Trp53 | stopgain | 62 | 55 | 0.53 |
| AOC11.9 | 11 | 79448137 | 79448137 | A | T | exonic | Nf1 | nonsynonymous SNV | 47 | 71 | 0.40 |
| AOC11.9 | 11 | 98427069 | 98427069 | A | T | exonic | Erbb2 | nonsynonymous SNV | 20 | 30 | 0.40 |
| AOC11.9 | 12 | 111261690 | 111261690 | A | T | exonic | Traf3 | nonsynonymous SNV | 33 | 42 | 0.44 |
| AOC11.9 | 2 | 26467981 | 26467981 | C | A | exonic | Notch1 | nonsynonymous SNV | 9 | 24 | 0.27 |
| AOC11.9 | 2 | 26470928 | 26470928 | G | T | exonic | Notch1 | nonsynonymous SNV | 17 | 7 | 0.71 |
| AOC11.9 | 3 | 34651294 | 34651294 | A | T | exonic | Sox2 | nonsynonymous SNV | 19 | 17 | 0.53 |
| AOC11.9 | 3 | 98111651 | 98111651 | G | T | exonic | Notch2 | stopgain | 28 | 8 | 0.78 |
| AOC11.9 | 3 | 98115238 | 98115238 | A | G | exonic | Notch2 | nonsynonymous SNV | 37 | 72 | 0.34 |
| AOC11.9 | 4 | 141322395 | 141322395 | G | A | exonic | Epha2 | nonsynonymous SNV | 26 | 30 | 0.46 |
| AOC11.9 | 7 | 130261767 | 130261767 | T | A | exonic | Fgfr2 | nonsynonymous SNV | 16 | 13 | 0.55 |
| AOC11.9 | 7 | 141192550 | 141192550 | T | A | exonic | Hras | nonsynonymous SNV | 31 | 22 | 0.58 |
| AOC11.9 | 8 | 45040766 | 45040766 | A | T | exonic | Fat1 | nonsynonymous SNV | 23 | 31 | 0.43 |
| AOC11.LN.7 | 11 | 69588505 | 69588505 | G | A | exonic | Trp53 | nonsynonymous SNV | 45 | 50 | 0.47 |
| AOC11.LN.7 | 11 | 69588676 | 69588676 | G | T | exonic | Trp53 | stopgain | 90 | 88 | 0.51 |
| AOC11.LN.7 | 11 | 79448137 | 79448137 | A | T | exonic | Nf1 | nonsynonymous SNV | 86 | 82 | 0.51 |
| AOC11.LN.7 | 11 | 98427069 | 98427069 | A | T | exonic | Erbb2 | nonsynonymous SNV | 48 | 42 | 0.53 |
| AOC11.LN.7 | 12 | 111261690 | 111261690 | A | T | exonic | Traf3 | nonsynonymous SNV | 57 | 53 | 0.52 |
| AOC11.LN.7 | 2 | 26467981 | 26467981 | C | A | exonic | Notch1 | nonsynonymous SNV | 25 | 22 | 0.53 |
| AOC11.LN.7 | 2 | 26470928 | 26470928 | G | T | exonic | Notch1 | nonsynonymous SNV | 25 | 9 | 0.74 |
| AOC11.LN.7 | 3 | 34651294 | 34651294 | A | T | exonic | Sox2 | nonsynonymous SNV | 27 | 18 | 0.60 |
| AOC11.LN.7 | 3 | 98111651 | 98111651 | G | T | exonic | Notch2 | stopgain | 31 | 39 | 0.44 |
| AOC11.LN.7 | 3 | 98115238 | 98115238 | A | G | exonic | Notch2 | nonsynonymous SNV | 75 | 61 | 0.55 |
| AOC11.LN.7 | 4 | 141322395 | 141322395 | G | A | exonic | Epha2 | nonsynonymous SNV | 66 | 32 | 0.67 |
| AOC11.LN.7 | 7 | 130261767 | 130261767 | T | A | exonic | Fgfr2 | nonsynonymous SNV | 27 | 38 | 0.42 |
| AOC11.LN.7 | 7 | 141192550 | 141192550 | T | A | exonic | Hras | nonsynonymous SNV | 10 | 59 | 0.14 |
| AOC11.LN.7 | 8 | 45040766 | 45040766 | A | T | exonic | Fat1 | nonsynonymous SNV | 31 | 37 | 0.46 |
| AOC3.3 | 11 | 69588425 | 69588425 | G | A | exonic | Trp53 | stopgain | 37 | 47 | 0.44 |
| AOC3.3 | 15 | 98843226 | 98843226 | C | A | exonic | Kmt2d | stopgain | 24 | 21 | 0.53 |
| AOC3.3 | 4 | 89276922 | 89276922 | A | T | exonic | Cdkn2a | nonsynonymous SNV | 20 | 16 | 0.56 |
| AOC3.3 | 4 | 141308673 | 141308673 | A | T | exonic | Epha2 | nonsynonymous SNV | 23 | 40 | 0.37 |
| AOC3.3 | 8 | 25557760 | 25557760 | G | C | exonic | Fgfr1 | nonsynonymous SNV | 94 | 74 | 0.56 |
| AOC3.3 | 8 | 44989044 | 44989044 | A | T | exonic | Fat1 | nonsynonymous SNV | 53 | 61 | 0.46 |
| AOC3.3 | 8 | 45017892 | 45017892 | A | T | exonic | Fat1 | nonsynonymous SNV | 27 | 28 | 0.49 |
| AOC3.3 | 8 | 45024075 | 45024075 | A | T | exonic | Fat1 | nonsynonymous SNV | 58 | 22 | 0.73 |
| AOC3.3 | 9 | 53506620 | 53506620 | T | A | exonic | Atm | stopgain | 37 | 51 | 0.42 |
| AOC3.LN.3 | 11 | 69588425 | 69588425 | G | A | exonic | Trp53 | stopgain | 32 | 35 | 0.48 |
| AOC3.LN.3 | 15 | 98843226 | 98843226 | C | A | exonic | Kmt2d | stopgain | 28 | 21 | 0.57 |
| AOC3.LN.3 | 4 | 89276922 | 89276922 | A | T | exonic | Cdkn2a | nonsynonymous SNV | 27 | 19 | 0.59 |
| AOC3.LN.3 | 4 | 141308673 | 141308673 | A | T | exonic | Epha2 | nonsynonymous SNV | 34 | 29 | 0.54 |
| AOC3.LN.3 | 8 | 25557760 | 25557760 | G | C | exonic | Fgfr1 | nonsynonymous SNV | 115 | 64 | 0.64 |
| AOC3.LN.3 | 8 | 44989044 | 44989044 | A | T | exonic | Fat1 | nonsynonymous SNV | 46 | 56 | 0.45 |
| AOC3.LN.3 | 8 | 45017892 | 45017892 | A | T | exonic | Fat1 | nonsynonymous SNV | 34 | 40 | 0.46 |
| AOC3.LN.3 | 9 | 53506620 | 53506620 | T | A | exonic | Atm | stopgain | 41 | 35 | 0.54 |
